# Comparison of four commercial devices for RapidArc and sliding window IMRT QA

**DOI:** 10.1120/jacmp.v12i2.3367

**Published:** 2011-04-04

**Authors:** Varatharaj Chandraraj, Sotirios Stathakis, Ravikumar Manickam, Carlos Esquivel, Sanjay S. Supe, Nikos Papanikolaou

**Affiliations:** ^1^ Department of Radiation Oncology, CTRC The University of Texas Health Science Center at San Antonio TX 78229 USA; ^2^ Department of Radiation Physics Kidwai Memorial Institute of Oncology Bangalore 560029 India

**Keywords:** IMRT, quality assurance, photons, film dosimetry, RapidArc

## Abstract

For intensity‐modulated radiation therapy, evaluation of the measured dose against the treatment planning calculated dose is essential in the context of patient‐specific quality assurance. The complexity of volumetric arc radiotherapy delivery attributed to its dynamic and synchronization nature require new methods and potentially new tools for the quality assurance of such techniques. In the present study, we evaluated and compared the dosimetric performance of EDR2 film and three other commercially available quality assurance devices: IBA I'MatriXX array, PTW Seven29 array and the Delta^4^ array. The evaluation of these dosimetric systems was performed for RapidArc and IMRT deliveries using a Varian NovalisTX linear accelerator. The plans were generated using the Varian Eclipse treatment planning system. Our results showed that all four QA techniques yield equivalent results. All patient QAs passed our institutional clinical criteria of gamma index based on a 3% dose difference and 3 mm distance to agreement. In addition, the Bland‐Altman analysis was performed which showed that all the calculated gamma values of all three QA devices were within 5% from those of the film. The results showed that the four QA systems used in this patient‐specific IMRT QA analysis are equivalent. We concluded that the dosimetric systems under investigation can be used interchangeably for routine patient specific QA.

PACS numbers: 87.55.Qr, 87.56.Fc

## I. INTRODUCTION

The aim of radiotherapy is to maximize dose to the tumor while, at the same time, minimize the dose to the organs at risk. One of the methods to realize this goal is by modulating the intensity within each beam, also known as intensity‐modulated radiation therapy (IMRT). IMRT is an extension of 3D conformal radiation therapy (3DCRT) in which only the beam apertures conform to the projected shape of the target.^(1)^

The delivery of IMRT is possible using a computer‐controlled multileaf collimator (MLC). IMRT is most commonly delivered either using the step and shoot^(2)^ or the sliding window^(3)^ method. The nonuniform intensity of each beam is produced by a superposition of smaller fields within each field (segments), each one with different shape, and monitor units. In the step‐and‐shoot approach, the MLC shape of the segments remains unchanged while the beam is on and changes while the beam is off. During IMRT delivery using the sliding window technique, each leaf pair moves continuously and unidirectionally at a variable speed while the beam is on. Any shape of intensity profile can be obtained by controlling the leaf movement, subject to the mechanical constraints such as leaf width, maximum speed and field size associated with a specific MLC system.

A more complex method of delivering nonuniform intensity beams is by introducing gantry rotation while modulating the intensity of the field. This technique is called intensity‐modulated arc herapy^(4)^ (IMAT). If the complexity of the delivery includes modulation of the dose rate, it is referred to as volumetric modulated arc therapy^(5)^ (VMAT). Recently, RapidArc (Varian Medical Systems, Palo Alto, CA) has become available for the treatment planning and delivery of the arc‐dynamic IMRT. It incorporates capabilities such as variable dose‐rate, variable gantry speed, and fast dynamic multileaf collimators (DMLC), to optimize dose conformity, delivery efficiency, accuracy and reliability.^(6)^ RapidArc is regarded as a complex treatment because the leaves of the MLC are continuously moving, and the gantry speed and dose rate are variably modulated. Because of the increased degrees of modulation present in RapidArc delivery, a robust QA program is necessary, allowing for an efficient and effective way to perform quality assurance on a routine basis.^(7)^

A hypothetical ideal dosimeter for IMRT QA was described by Nelms et al.^(8)^ It consists of very small (submillimeter) isotropic absolute dose detectors arranged in a high‐density three‐dimensional array in a water‐equivalent phantom. Such detector system should have linear response, reproducibility and energy independence. Such an idealistic IMRT dosimeter has not been built to date.^(9)^ Since the introduction of IMRT, film, ion chambers,^(10)^ a 2D array of detectors^(11)^ and gel dosimetry^(12)^ have been used specifically for the pretreatment verification of rotational radiotherapy.

Film dosimetry has been widely adopted for IMRT QA due to its high spatial resolution; unfortunately, film is a time‐consuming procedure and requires great care if it is used as an absolute dosimeter.^(13)^ Often, an independent ionometric measurement is necessary to assess the absolute dose agreement between the plan and the measurement. Arrays of detectors are now replacing films for routine IMRT QA, and require very simple setup and verification procedures.

Commercially available 2D array detector devices can be based on either diode or ionization chamber construction.^(^ ^14^ ^,^ ^15^ ^)^ Regardless of the detector type used, they have excellent characteristics in terms of linearity, repeatability and independence from dose rate effects. However, they have limited spatial resolution due to the discrete placement and physical separation of each detector as compared to film. Poppe et al.^(16)^ analyzed the influence of those parameters on accuracy of IMRT dose measurements and concluded that dose variations in realistic IMRT dose distributions contain very little, if any, spatial frequency components above $0.1{\text{mm}}^{-1}$. Spezi et al.^(17)^ showed that the PTW 2D‐ARRAY seven29 can detect leaf positional errors down to 1 mm. Similar findings have been reported for the MatriXX^(18)^ and Delta^4^ arrays.^(9)^

In this study, we aim to evaluate and compare the dosimetric performance of EDR2 film and three other commercial QA devices: IBA I'MatriXX (IBA dosimetry, GmbH, Germany), PTW seven29 array (PTW Freiburg GmbH, Germany) and Delta^4^ array (ScandiDos AB, Uppsala, Sweden). The evaluation of these dosimetric systems was applied to RapidArc and IMRT deliveries using a Varian NovalisTX linear accelerator with plans generated using the Varian Eclipse (ver. 8.6) treatment planning system (TPS).

## II. MATERIALS AND METHODS

### A. Planning and delivery

Fifteen (n=15) consecutive RapidArc plans and ten (n=10) IMRT plans were used for this study. Treatment site selection and MUs for each RapidArc plan and for each of the sliding window IMRT plans are shown in Table 1. All patient plans were optimized using the Eclipse TPS. A QA plan for each of the four QA devices was calculated for each of the patient plans. The measurement geometries include a film sandwiched in an acrylic phantom, the IBA MultiCube (IBA dosimetry, GmbH, Germany) with the MatriXX, the PTW OCTAVIUS phantom (PTW Freiburg GmbH, Germany) with the PTW seven29 array, and the ScandiDos Delta^4^ device. The verification plans were calculated and delivered using the actual planned gantry and collimator angles, respectively. The treatment couch was also included in the calculation of the verification plans. For the comparison between measurements and calculations, the analysis was performed only for the cumulative dose from all beams in a plan.

**Table 1:** Treatment site selection and MU for each plan for the RapidArc and IMRT plans.

*RapidArc*		*IMRT*		
*SITE(Dose)*	*MU*	*SITE(Dose)*	*MU*	*Total MU*
Prostate(2.0Gy)	871	Prostate(2.0Gy)	168,145,168,176,140	797
Prostate(2.7Gy)	1010	Prostate(2.0Gy)	147,132,135,141,114	669
Prostate(2.0Gy)	512	Prostate(2.0Gy)	144,135,136,139,154	708
H&N(2.0Gy)	621	Prostate(2.0Gy)	105,103,127,124,103	562
Prostate boost (2.0Gy)	816	Prostate(2.0Gy)	148,112,175,126,124	685
Brain(2.0Gy)	369	H&N(2.0Gy)	116,85,59,78,99,77,89	603
Brain(15.0Gy)	5134	Brain(2.0)	63,76,79,66,86,87,86	543
Prostate(2.0Gy)	403	Prostate(2.0Gy)	106,94,93,111,97,90,101	692
Spine(2.0Gy)	892	Liver(1.8Gy)	81,96,158,124,89,85,102	735
Lung(5.0Gy)	961	Lung(1.8Gy)	100,82,84,89,103,114,84	656
Prostate boost (2.0Gy)	(408),(344)			
Prostate(2.0Gy)	(240),(277)			
Lung(15.0Gy)	(2396),(919),(918),(749)			
Liver(10.0Gy)	(1886),(216)			
Prostate(2.0Gy)	602			

All plans were delivered on a Varian NovalisTX linear accelerator equipped with a high‐definition multileaf collimator (HDMLC). Six and 10 MV beams were used, depending on the plan. The license to deliver extended monitor units was enabled in our linac allowing us to treat with up to 7000 monitor units per beam. For the SBRT RapidArc plans with prescription doses of 15 Gy per fraction, the monitor units and the dose rate were scaled down for the film measurements, to allow for a meaningful measurement without saturating the film.

### B. EDR2 film measurements

**Figure 1:**
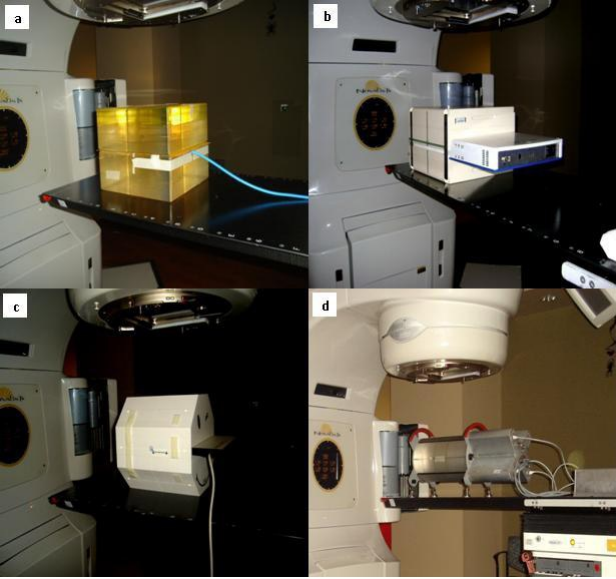
Setup arrangement for measurements using: (a) EDR 2 film, (b) I'MatriXX, (c) PTW seven29 array, and (4) Delta^4^ array.

EDR2 film (Kodak, Rochester, NY) is a slow speed, fine grain film. It uses fine monodispersed grain cubic microcrystals. Double emulsion layers are coated on a 0.18 mm base which allows processing of film in a conventional rapid film processor.^(19)^ A Kodak automatic processor was used for processing the exposed EDR2 films. The EDR2 film measurements were performed by placing the films in the middle of an acrylic phantom produced in‐house (Fig. 1(a) The developed films were scanned using a VXR‐16 Dosimetry Pro scanner (Vidar Systems Corp., Herndon, VA). The IMRT analysis was carried out using the RIT 113 version 5.2 (Radiological Imaging Technology, Inc., Colorado Springs, CO) software. The analysis involved the registration of the film to the same geometrical origin as for the RTP calculated dose plane. The film measurement and the calculated planar dose were normalized to an area with a uniform dose in the high‐dose region. A film calibration prior to measurements was completed to allow us to convert the film optical density to dose. The calibration used a film from the same film batch that was exposed to a step wedge at varying dose levels ranging from 10 to 400 cGy.

### C. I'mRT MatriXX with MultiCube phantom

The IBA I'mRT MatriXX is a two‐dimensional array of 1020 vented ion chambers, arranged in a 32×32 grid. Each chamber volume is 0.08 cm^3^ with a height of 5.0 mm and diameter of 4.5 mm with equivalent water thickness on the front side of the detectors of 3.6 mm. The maximum dose rate detectable by the detectors is 5.0 Gy/min and minimum detectable dose rate is 0.1 Gy/min. The bias voltage required for the MatriXX system is 500±30 V and is set by the manufacturer. The maximum field of view is $24\times 24\;{\text{cm}}^{2}$ and the center‐to‐center separation between the chambers is 7.62 mm. All 25 verification plans were delivered to the MatriXX, which was inserted into the IBA MultiCube virtual water phantom. The size of MultiCube is 31.4 cm (L)×34 cm (W)×34 cm (H) and the approximate weight is 33.0 kg. A 15‐minute warm‐up time and a minimum of 500 MU of pre‐irradiation before each use were performed as recommended by the manufacturer. An absolute calibration of the array detectors was performed according to the manufacturer recommended procedures. The procedure involves the measurement of the absolute dose for each photon beam energy using a calibrated ionization chamber inside the MultiCube at the level of the MatriXX center. The measured absolute dose, temperature and pressure are entered in the OmniPro software prior to the delivery of the same MU. An internal calibration factor (kuser) is calculated based on the measurement values obtained from the four center MatriXX chambers. (Figure 1b) shows the setup arrangement for the measurements with the MatriXX.

### D. PTW seven29 2D‐ARRAY with OCTAVIUS phantom

The PTW seven29 2D‐ARRAY consists of 729 vented plane‐parallel ionization chambers with a $0.6{g/\text{cm}}^{2}$ graphite wall arranged in a 27×27 matrix covering an area of 27 cm×27 cm. Each single chamber is air‐filled with a cross section of 5 mm×5 mm and height of 5 mm. The chambers are separated from each other by 5 mm. The distance between the centers of adjacent chambers is 10 mm. The 2D array surrounding material is made up of polymethyl methycrylate (PMMA). The measuring system consists of the chamber array itself (which also accommodates part of the electronic devices), the array interface, and a data acquisition board for the personal computer. A dedicated phantom for the QA of rotational treatments focusing primarily on the use of the seven29 2D ion chamber array, called OCTAVIUS was used during measurements. OCTAVIUS is made of polystyrene (physical density $1.04\;{g/\text{cm}}^{3}$, relative electron density 1.00), is 32 cm wide and has a length of 32 cm. A $30\times 30\times 2.2\;{\text{cm}}^{3}$ central cavity allows the user to insert the 2D ion chamber array into the phantom. The position of the cavity is such that when the 2D array is inserted, the plane through the middle of the ion chambers goes through the center of the phantom. The measurement ranges for PTW seven29 as specified by manufacturer are 200 mGy–1000 Gy and 500 mGy ${\text{min}}^{-1}$ to 8 Gy ${\text{min}}^{-1}$. The 2D array is calibrated for absolute dosimetry in a ${}^{60}\text{Co}$ photon beam at the PTW secondary standard dosimetry laboratory. An on‐site output factor correcting for the daily output fluctuations of the beam can be measured and used by the detector acquisition software. In this work, the detector array was used in absolute dose measuring mode and dose values were corrected for daily variation of linac output.

### E. ScandiDos Delta4 phantom

The Delta^4^ device consists of 1069 p‐type Silicon diodes arranged in a matrix along two orthogonal planes. Each p‐type diode has a cylindrical sensitive volume with a 0.78 mm^2^ area and a thickness of 0.05 mm. The detectors are spaced at 0.5 cm intervals in the central 6 cm×6 cm area and at 1 cm intervals outside of this area, and they cover an area of 20 cm×20 cm. The detector planes are placed in an acrylic cylindrical phantom 22 cm in diameter and 40 cm in length. The crossed planes are achieved by means of a main detector board which spans through the entire diameter of the phantom, and two wing detector boards which are separated to allow the main detector board to be positioned between them.^(20)^ With this device the dose can be measured to as low as 1 mGy. Gantry angle position is independently recorded by means of an inclinometer attached to the gantry. This allows the device to identify which control point of a dynamic arc delivery is being delivered, so that the measured dose can be associated with this control point and the appropriate correction for gantry angle is applied. Multichannel electrometers are located at the ends of the detector planes in an integrated module. A coaxial cable is run into the treatment room to provide beam synchronization pulses from the accelerator to the electrometers, and a CAT‐5 cable is run out of the room to transfer the data to the control computer. The measured data are synchronized with the accelerator pulses and stored on a pulse‐by‐pulse basis, allowing segment‐by‐segment analysis and 4D treatment QA. Calibration of the detector arrays involves two steps: absolute calibration of the central detector, and relative calibration of all the detectors. The absolute calibration needs to be done for each beam energy of the linear accelerator. The relative calibration only needs to be done once. Both of these calibrations are done with detectors removed from the cylindrical phantom and placed in a rectangular acrylic slab phantom. Absolute dose is determined for a $10\times 10\;{\text{cm}}^{2}$ reference condition using an ion chamber in the same phantom that will be used for normalizing the detector arrays. The ionization chamber is replaced by the detector arrays, and the absolute sensitivity of the central detector is determined. The relative sensitivity is determined in the same phantom but with a field size large enough to encompass the detector arrays. The cross‐calibration process involves irradiating and then translating the arrays. This is repeated seven times with translations in both directions perpendicular to the beam in order to cross‐calibrate the detectors in a stable beam.^(20)^ The device was allowed to warm up for 30 minutes prior to use as shown in Fig. 1(d).

### F. Evaluation metrics

In addition to the qualitative evaluation of multiple dose profiles and the isodose distribution comparison, the γ‐index^(21)^ (as implemented by each manufacturer) was used for quantifying the agreement between calculations and measurements. The γ‐index criteria used in our analysis was 3% dose differences and 3 mm distance to agreement. The γ‐index is formulated such that a favorable comparison will yield a γ‐value less than one, where both the dose difference and the distance to agreement (DTA) are better than the user set threshold value. For this study, measurements for plans with 90% of gamma points less than 1 were considered acceptable. Prior to analysis of the measurements, tests including simple geometries (square fields and wedged fields) were calculated and delivered to each detector in order to test the gamma calculation for each software. The results were within less than 1% and, hence, we decided to use each software's gamma index calculation method for its respective measurement.

All measurements were compared against calculated doses, first by taking into account all points in the measurement planes, and then by applying a threshold value where the points that received less than 20% of the maximum dose were excluded from the gamma index calculation.

In order to evaluate the results obtained from each device, we considered the film to be the gold standard. We then used the Bland‐Altman statistical method^(22)^ to compare the performance of the other detectors against the film. Furthermore, statistics (mean, standard deviation, etc.) within each set of measurements were calculated and evaluated.

## III. RESULTS

**Figure 2:**
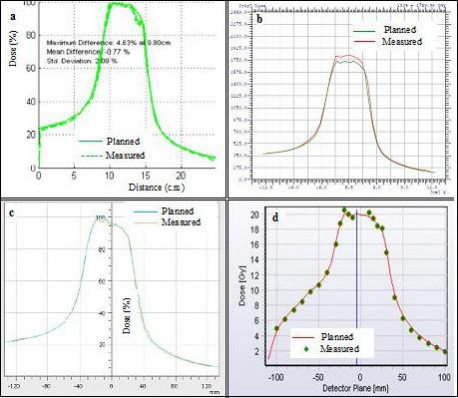
Comparisons of measured and planned horizontal profiles for a lung RapidArc case using: (a) film, (b) I'MatriXX, (c) PTW seven29 array, and (d) Delta^4^ array.

**Figure 3:**
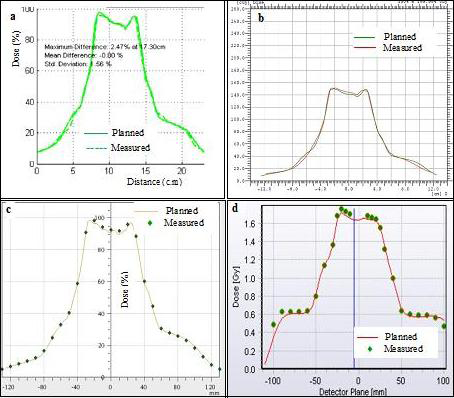
Comparisons of measured and planned horizontal profiles for a head and neck sliding window IMRT case using: (a) film, (b) I'MatriXX, (c) PTW seven29 array, and (d) Delta^4^ array.

**Figure 4:**
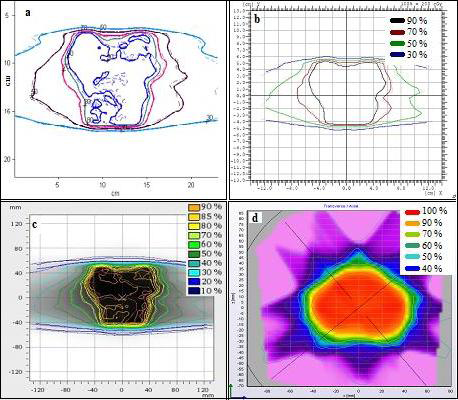
Comparisons of measured (solid lines) and planned (dotted lines) isodose distributions for a prostate RapidArc case using: (a) film, (b) I'MatriXX, (c) PTW seven29 array, and (d) Delta^4^ array.

**Figure 5:**
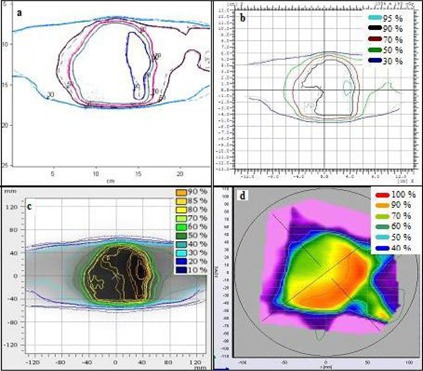
Comparisons of measured (solid lines) and planned (dotted lines) isodose distributions for a liver sliding window IMRT case using: (a) film, (b) I'MatriXX, (c) PTW seven29 array, and (d) Delta^4^ array.

Figure 2 shows a typical comparison between measured and planned horizontal profiles for a lung RapidArc case using the four different detectors. The equivalent results for a head and neck sliding window IMRT case are shown in Fig. 3. Figures 4 and 5 show the measured and planned isodose distribution comparisons with the four different detectors for a prostate RapidArc case and a liver case using sliding window IMRT, respectively. Similar results were obtained for the rest of the cases (not shown).

Table 2 shows the comparison of gamma values for the 15 RapidArc cases and Table 3 shows the comparison for the 10 sliding window IMRT QA plans. All 25 plans passed the gamma evaluation analysis when using all the points in the planar dose image for the evaluation. When the analysis was confined to those points in the dose image with dose of at least 20% of the maximum dose or higher, some plans did not pass the gamma analysis using our set criteria. In the case of RapidArc QA measurements, several film and one PTW seven29 measurements failed, while for the IMRT QA plans, two film and two I'MatriXX measurements failed. All of Delta^4^ measurements met the gamma criteria.

**Table 2:** Comparison of gamma values and the statistical parameters for RapidArc cases of four different detectors: for 0% of the normalized dose for all calculation points in the measurement plane(s), and for only those points that received at least 20% of the normalized dose.

*PLAN NO*	*SITE*	*FILM (0%)*	*FILM (20%)*	*MATRIX (0%)*	*MATRIX (20%)*	*PTW (0%)*	*PTW (20%)*	Δ*4 (0%)*	Δ*4 (20%)*	*Mean (0%)*	*Std. Dev. (0%)*	*Mean (20%)*	*Std. Dev. (20%)*
1	Brain	91.69	83.09	99.78	99.26	94.55	89.59	98.8	96.7	96.20	3.770	92.16	7.300
2	Brain	99.34	90.66	99.24	91.81	98.9	97.66	99.8	96.8	99.32	0.371	94.23	3.510
3	H&N	94.38	87.96	98.76	97.42	95.79	94.06	99	98.4	96.98	2.267	94.46	4.715
4	Liver	99.99	99.9	100	99.99	99.78	98.95	99.8	98.4	99.89	0.118	99.31	0.767
5	Lung	99.63	99.03	95.74	90.65	98.7	97.89	99.2	98.5	98.31	1.759	96.51	3.939
6	Lung	99.87	99.21	97.89	91.02	99.95	99.7	98.1	95.1	98.95	1.109	96.25	4.055
7	Prostate	94.95	82.49	98.33	95.14	96.05	97.52	97.2	93.9	96.63	1.457	92.26	6.685
8	Prostate	98.79	97.11	97.89	95.84	97.94	97.85	98.1	96.6	98.18	0.416	96.85	0.846
9	Prostate	94.24	90.46	96.18	92.41	96.78	94.78	98.1	96.6	96.32	1.604	93.56	2.687
10	Prostate	93.98	82.57	97.5	93.93	96.2	92.19	98.4	96.8	96.52	1.919	91.37	6.168
11	Prostate	98.37	96.77	98.42	95.95	97.81	92.83	96.5	92	97.77	0.893	94.38	2.326
12	Prostate	99.11	97.84	95.12	94.81	98.42	97.99	97.9	95.9	97.63	1.749	96.63	1.544
13	Prostate	92.93	82.2	96.71	92.71	99.45	97.59	97.4	94.1	96.62	2.72	91.65	6.626
14	Prostate	99.23	97.99	98.91	97.63	99.78	98.95	98.9	97.8	99.20	0.412	98.09	0.590
15	Spine	96.76	88.74	96.22	90.29	98.72	98.46	99.3	97.2	97.75	1.490	93.67	4.869
	Average	96.884	91.735	97.78	94.591	97.922	96.401	98.43	96.32	97.755	2.105	94.761	2.183

**Table 3:** Comparison of gamma values the statistical parameters for sliding window IMRT cases of four different detectors: for 0% of the normalized dose for all calculation points in the measurement plane(s), and for only those points that received at least 20% of the normalized dose.

*PLAN NO*	*SITE*	*FILM (0%)*	*FILM (20%)*	*MATRIX (0%)*	*MATRIX (20%)*	*PTW (0%)*	*PTW (20%)*	Δ*4 (0%)*	Δ*4 (20%)*	*Mean (0%)*	*Std. Dev. (0%)*	*Mean (20%)*	*Std. Dev. (20%)*
1	Brain	98.24	94.25	99.73	99.28	96	96.84	98.9	97.7	98.21	1.599	91.41	7.790
2	H&N	98.21	93.87	98.98	97.75	96.96	94.45	98.7	96.9	98.21	0.893	94.23	3.510
3	Liver	97.32	93.97	97.3	95.22	96.19	95.07	97.6	95.7	97.10	0.623	94.46	4.715
4	Lung	93.62	84.73	98.89	99.07	97.47	93.55	97.3	95.3	96.82	2.249	99.31	0.767
5	Prostate	99.55	98.52	98.02	94.62	97.49	95.84	99.2	98.2	98.565	0.970	96.51	3.939
6	Prostate	94.92	87.23	96.55	93.18	98.13	97.63	96.9	94.5	96.625	1.323	96.25	4.055
7	Prostate	96.72	91.02	93.85	87.53	96.77	96.86	96.6	94	95.985	1.425	92.26	6.685
8	Prostate	98.47	95.43	99.05	98.69	97.74	96.21	97.3	94.8	98.14	0.775	96.85	0.846
9	Prostate	97.94	91.14	96.17	88.51	97.88	95.29	98.4	96.2	97.59	0.979	93.56	2.687
10	Prostate	99.63	98.19	98.19	94.39	99.96	98.55	97.9	94.9	98.92	1.026	91.37	6.168
	Average	97.462	92.835	97.673	94.824	97.459	96.029	97.88	95.82	97.618	1.186	94.62	4.116

**Figure 6:**
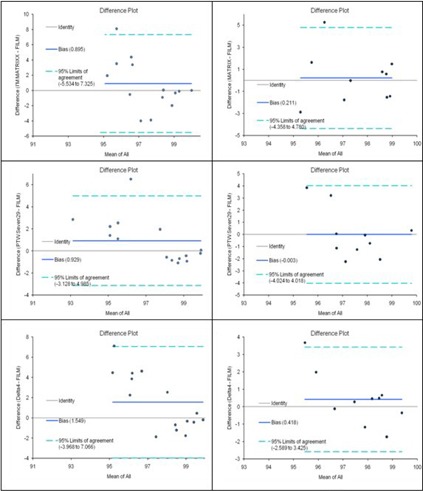
Bland‐Altman graphs of the agreement between film and the three detector array measurements.

On average, all the QA devices produced very similar results. When all points were included in the calculation of the gamma index, minimal differences were observed. This was further supported by the Bland‐Altman analysis that showed that all the calculated gamma values of all the detectors were within 5% from those of the film. (Table 4, Fig. 6).

**Table 4:** Statistical metrics from Bland‐Altman analysis of the measurements.

	*All Points Included*			*Points Above 20% of Maximum Dose*		
	*MatriXX*	*PTW*	${\text{Delta}}^{4}$	*MatriXX*	*PTW*	${\text{Delta}}^{4}$
Bias	−0.90	−1.04	1.55	−0.21	0.00	−0.428
SD of Bias	3.28	2.00	2.81	2.33	2.05	1.53
95% Limits	−7.33	−4.95	−7.07	−4.78	−4.02	−3.42
Agreement	5.53	2.88	3.97	4.34	4.02	2.59

## IV. DISCUSSION

Our results show that, overall, minimal differences exist between the four methods of IMRT plans dose verification. Film dosimetry is a well established method for verifying two‐dimensional IMRT dose distributions due to its high spatial resolution. On the other hand, film dosimetry dependents on photon energy and dose rate.^(23)^ In general, film dosimetry is a time‐ and material‐consuming method that requires well‐controlled chemistry, especially when absolute dosimetry is necessary.

It should be noted that the measurements were performed without applying any correction factors for angular dependence. The angular dependence of the devices in IMRT and VMAT patient‐specific QA has been reported to be minimal when the measurements from all angles are summed.^(^ ^8^ ^,^ ^9^ ^,^ ^11^ ^,^ ^15^ ^–^ ^18^ ^,^ ^24^ ^)^

All methods used for the measurements taken were subject to similar setup uncertainties. Daily linac output variations (± 0.5%) were only included in the case of film and PTW measurements. For the film, the output variations were included in the calibration curve and, for the PTW array, in the daily output correction measurement. Overall, the impact of the daily output variation did not have a significant impact on the measurements.

When stricter gamma index criteria were used, some of the measured planar doses failed to pass the tolerance of 90% of points with less than 1.0 gamma index. In the case of the film measurement, setup and registration uncertainties were primarily responsible for this discrepancy. The PTW and the MatriXX discrepancies are attributed to setup error and the inherent low resolution of such devices.

The statistical analysis performed showed that all the measurement methods can be used interchangeably, better than 95% of the time. Due to the improved efficiency and ease of use of the 2D arrays, they can be used in lieu of film and with minimal calibration requirements.^(17)^

Bedford et al.^(15)^ reported similar results on a comparison between film measurements and the Delta^4^. They reported that, from the gamma values, Delta^4^ shows slightly better agreement between measured and calculated doses than the film in a cuboid phantom. The reason could be due to the fact that the Delta^4^ measurements are absolute, whereas film is used as a relative dosimeter. Consequently, an absolute dose difference manifests itself in the Delta^4^ gamma, whereas such a difference is typically removed in the film through normalization. On the other hand, the Delta^4^ is not susceptible to errors introduced during film processing. Moreover, Delta^4^ uses a three‐dimensional gamma evaluation, which is less sensitive when compared to the two‐dimensional one used for film.

## V. CONCLUSIONS

In the present study, four different methods for patient specific RapidArc and IMRT QA were evaluated. The results showed that the differences between the detectors are insignificant. All patient QAs passed the criteria of gamma index values of 3% dose difference and 3 mm DTA. We conclude that the dosimetric systems under investigation can be used interchangeably for routine patient specific QA.

## ACKNOWLEDGMENTS

This work has been supported by a UICC International Cancer Technology Transfer Fellowship, and by funds from the National Cancer Institute, National Institutes of Health under Contract NO2‐CO‐41101.
